# Cross-reactivity between avian influenza A (H7N9) virus and divergent H7 subtypic- and heterosubtypic influenza A viruses

**DOI:** 10.1038/srep22045

**Published:** 2016-02-24

**Authors:** Li Guo, Dayan Wang, Hongli Zhou, Chao Wu, Xin Gao, Yan Xiao, Lili Ren, Gláucia Paranhos-Baccalà, Yuelong Shu, Qi Jin, Jianwei Wang

**Affiliations:** 1 MOH Key Laboratory of Systems Biology of Pathogens and Christophe Mérieux Laboratory, IPB, CAMS-Fondation Mérieux, Institute of Pathogen Biology (IPB), Chinese Academy of Medical Sciences (CAMS) & Peking Union Medical College, Beijing, P. R. China; 2 Collaborative Innovation Center for Diagnosis and Treatment of Infectious Diseases, Hangzhou, P. R. China; 3 Institute of Viral Disease Control and Prevention, Chinese Center for Disease Control and Prevention, Beijing, P. R.China; 4 Fondation Mérieux, IRF 128 BioSciences Lyon-Gerland, 69365 Lyon, France

## Abstract

The number of human avian H7N9 influenza infections has been increasing in China. Understanding their antigenic and serologic relationships is crucial for developing diagnostic tools and vaccines. Here, we evaluated the cross-reactivities and neutralizing activities among H7 subtype influenza viruses and between H7N9 and heterosubtype influenza A viruses. We found strong cross-reactivities between H7N9 and divergent H7 subtypic viruses, including H7N2, H7N3, and H7N7. Antisera against H7N2, H7N3, and H7N7 could also effectively neutralize two distinct H7N9 strains. Two-way cross-reactivities exist within group 2, including H3 and H4, whereas one-way cross-reactivities were found across other groups, including H1, H10, H9, and H13. Our data indicate that the hemaglutinins from divergent H7 subtypes may facilitate the development of vaccines for distinct H7N9 infections. Moreover, serologic diagnoses for H7N9 infections need to consider possible interference from the cross-reactivity of H7N9 with other subtype influenza viruses.

In March 2013, a novel avian influenza A(H7N9) virus (hereafter H7N9) was identified in China^1^. Since then, the number of human infections with H7N9 has been steadily increasing in the country. As of May 3^rd^, 2015, 657 infected cases and 263 deaths have been reported (http://www.who.int/influenza/human_animal_interface/influenza_h7n9/en/). Most patients who are infected with the virus progress to pneumonia and acute respiratory distress syndrome, with a case fatality rate of approximately 40%^1^,^2^. H7N9 shows limited transmissibility in ferrets and guinea pigs but possesses amino acid changes that allow it to adapt to mammalian hosts, which raises the concern for pandemics in humans^3^,^4^,^5^,^6^. Before its emergence, there was no human immunity to this virus^7^,^8^. It is therefore urgently necessary to develop effective diagnostics and therapeutics as surveillance and control strategies against a potential outbreak of H7N9 disease.

Influenza A viruses are classified into subtypes based on the antigenicity and phylogenetics of their hemagglutinin (HA) and neuraminidase (NA)^9^. Thus far, 18 HA subtypes (H1–H18) and 11 NA subtypes (N1–N11) have been identified^10^. The HA subtypes can be further divided into two antigenically-distinct groups: group 1 (H1, H2, H5, H6, H8, H9, H11, H12, H13, H16, H17, and H18) and group 2 (H3, H4, H7, H10, H14, and H15) according to their phylogenetic relationships based on the amino acid sequences of HA^11^.

However, with the emergence of the H7N9 virus, the serologic and antigenic relationships between the novel virus and heterosubtypic influenza viruses are unclear. The fact that there are so many immunologically distinct influenza viruses illustrates the importance of comprehensively understanding serologic and antigenic relationships in order to track H7N9 in human populations rapidly and optimize the diagnostic tools and vaccines for H7N9.

Previous sporadic human infections with avian H7 virus strains have prompted preclinical and early clinical vaccine development^12^,^13^. However, it is not well understood whether divergent H7 subtypic and heterosubtypic influenza viruses have cross-neutralizing activity with the recently identified H7N9 strains. We have shown that there are cross-reactivities between seasonal influenza viruses (H3N2 and H1N1) and convalescent-phase sera of H7N9 virus-infected patients^7^. However, we could not confirm the presence of cross-reactivity using only the convalescent-phase sera of H7N9 virus-infected patients because preexisting antibodies to seasonal influenza viruses interfered with the results. To clarify the serologic and antigenic relationships between the H7N9 and divergent H7 subtypic- and heterosubtypic influenza viruses, we used two distinct H7N9 virus strains, HA proteins from three divergent H7 subtype and 13 heterosubtypic influenza viruses, and 12 immunized animal antisera against HA proteins of heterosubtypic A influenza viruses to evaluate the cross-reactivities and neutralizing activities among H7 subtype influenza viruses and between H7N9 and heterosubtype influenza viruses.

## Results

### Cross-reactivities within H7 subtype influenza viruses

**Figure 1:**
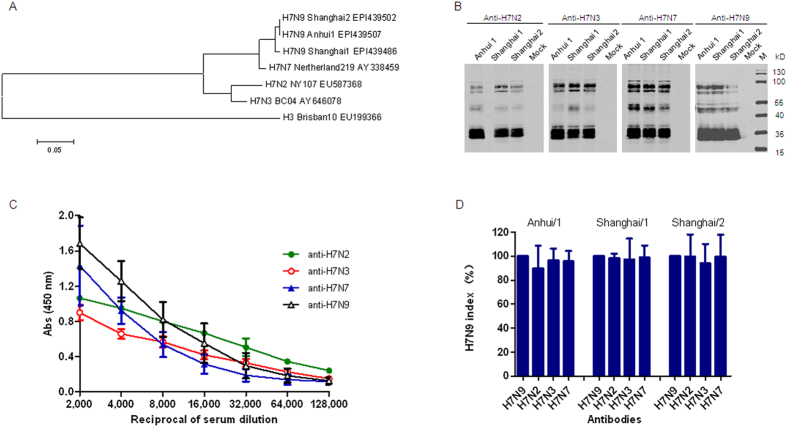
Cross-reactivities among H7 subtypes known to infect humans. (**A**) Phylogeny of H7 subtypes. Phylogenetic tree based on HA amino acid sequences of H7 subtypes generated using the Clustal W and MegAlign programs in the MEGA4.0 software package, including H7N9, H7N2, H7N3, H7N7, and H3N2. (**B**) Western blot analysis of lysates of three different preparations of MDCK cells infected with Anhui/1, Shanghai/1, and Shanghai/2 isolates. Isolates were harvested 48 h post infection at an MOI of 0.1. Blots were probed with the antisera against HA proteins of H7N9, H7N2, H7N3, and H7N7. (**C**) Cross-reactivities in ELISA. H7N9 HA expressed in insect cells was used as the antigen. Mouse antisera against H7N2, H7N3, and H7N7 were two-fold diluted from 1:2,000 to a final dilution of 1:128,000. Antisera against H7N9 HA were used as positive control. (**D**) Cross-reactivities in indirect immunofluorescence assays. Three different preparations of MDCK cells infected with Anhui/1, Shanghai/1, and Shanghai/2 at an MOI of 0.1 were fixed with 4% formaldehyde and probed with antisera against HA proteins of H7N2, H7N3, and H7N7 with a concentration of 0.5 μg/ml. Antisera against H7N9 HA were used as positive control. H7N9 index calculated by IFA data.

To determine the cross-reactivities within H7 subtype influenza viruses, we first analyzed the cross-reactivity between H7N9 and other H7 subtypes, such as H7N2, H7N3, and H7N7 viruses, which are known to infect human beings and are closely related to H7N9 (Fig. 1A). To this end, we expressed and purified trimeric HA proteins of H7N9, H7N2, H7N3, and H7N7 viruses. To verify the immunogenicity of these proteins, we immunized mice with purified HA proteins. After three rounds of immunization, specific IgG antibodies against HAs with titers of about 80,000 were elicited (Figure S1), indicating that these trimeric HA proteins are immunogenic in mice. We then used these murine antisera to perform Western blot analysis with the lysates from MDCK cells infected with H7N9 isolates. All of the antisera recognized the HA0, HA1, and HA2 bands of the Anhui/1, Shanghai/1, and Shanghai/2 isolates (Fig. 1B), suggesting that there is cross-reactivity between H7N9 and H7N2, H7N3, and H7N7.

To further evaluate the cross-reactivity within H7 subtypes, we performed an ELISA using the Anhui/1 HA as a coating antigen and measured its binding ability to antisera against HA proteins of H7N2, H7N3, and H7N7 using the anti-Anhui/1 HA antisera as a positive control. We found that Anhui/1 HA was recognized by divergent H7 subtype mouse polyclonal antibodies (Fig. 1C). Similar results were observed for HA proteins of H7N2, H7N3, and H7N7 with divergent H7 subtype mouse polyclonal antibodies (Figure S2). Indirect immunofluorescence assay (IFA) further showed that antisera against HA proteins of H7N2, H7N3, and H7N7 recognized HA proteins from MDCK cells infected with the Anhui/1, Shanghai/1, and Shanghai/2 isolates (Fig. 1D). Collectively, these data indicate strong two-way cross-reactivity within H7 subtype influenza viruses.

### Antigenic relationships between H7N9 and heterosubtypic influenza A viruses

**Figure 2:**
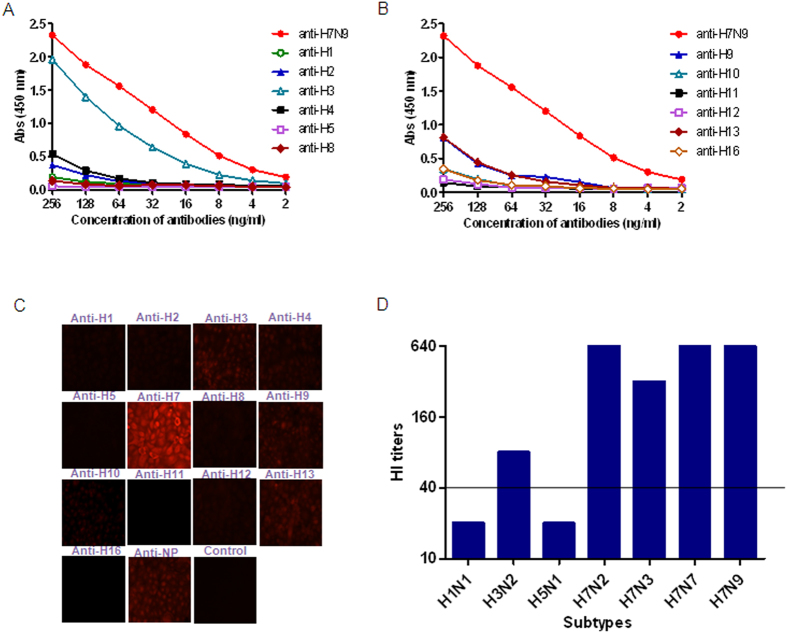
Cross-reactivity between H7N9 HA and antibodies against heterosubtypes of influenza A viruses. (**A,B**) Cross-reactivities in ELISA. ELISA tests were performed with H7N9 HA expressed in insect cells as coating antigen. Antisera against H1, H2, H3, H4, H5, and H8 (**A**), and H9, H10, H11, H12, H13, and H16 (**B**) were serially diluted starting at a dilution of 256 ng/ml to react with the coating antigens. Antisera against H7N9 HA were used as positive control. (**C**) Cross-reactivities in indirect immunofluorescence assays. MDCK cells infected with Anhui/1 at an MOI of 0.1 were fixed with 4% formaldehyde and probed with antisera against HA proteins of H1, H2, H3, H4, H5, H8, H9, H10, H11, H12, H13, and H16 with a concentration of 0.5 μg/ml. Antisera against H7N9 HA were used as positive control. (**D**) Cross-reactivities in Hemagglutination inhibition (HI) assays. The assays were carried out using the A/Anhui/1/2013 (H7N9) strain and antisera against whole virus of H1N1, H3N2, and H5N1 with antisera against H7N9-, H7N2-, H7N3-, and H7N7-HA as positive controls. Serum with titers >40 were considered HI-positive for H7N9 virus.

Next, we assessed the antigenic relationships between H7N9 and representative influenza virus HA proteins from groups 1 and 2 using enzyme-linked immunosorbent assay (ELISA), IFA, and Western blot analyses. Cross-reactivity was called only when detected by at least two different methods. The HA protein from H7N9 Anhui/1 and the highly specific antisera against 12 heterosubtypes of influenza viruses were used to perform ELISA analysis. We found that H7N9 HA was strongly recognized by rabbit antiserum against H3. It was also recognized by H4, but at a lower level (Fig. 2A). Both H3 and H4 belong to group 2. Unexpectedly, Anhui/1 HA also showed low level cross-reactivity with antisera against H9 and H13 (Fig. 2B), both of which belong to group 1.

Cross-reactivities were further verified by IFA using H7N9 virus-infected cells with a panel of antisera to HAs. The results were similar to those observed in the ELISA assay. MDCK cells infected with Anhui/1 bound at a medium level to antisera against H3 and H13 (Fig. 2C) and at a low level to antisera against H4, H9, H10, and H12 (Fig. 2C).

As the hemagglutination inhibition (HI) assay is widely used in influenza surveillance and diagnosis, we also used ferret antisera to perform HI assays, including antisera against whole H1N1, H3N2, and H5N1 viruses using mouse antisera against HA proteins of H7 subtypes as positive controls. We observed very strong cross-reactivity between the H7N9 virus and H7 subtype antisera. The HI titers were 640, 640, 320, and 640 for H7N9, H7N2, H7N3, and H7N7, respectively. Consistent with the ELISA, IFA, and Western blot analyses, cross-reactivity was observed between H7N9 and H3N2 (1:80) (Fig. 2D).

**Figure 3:**
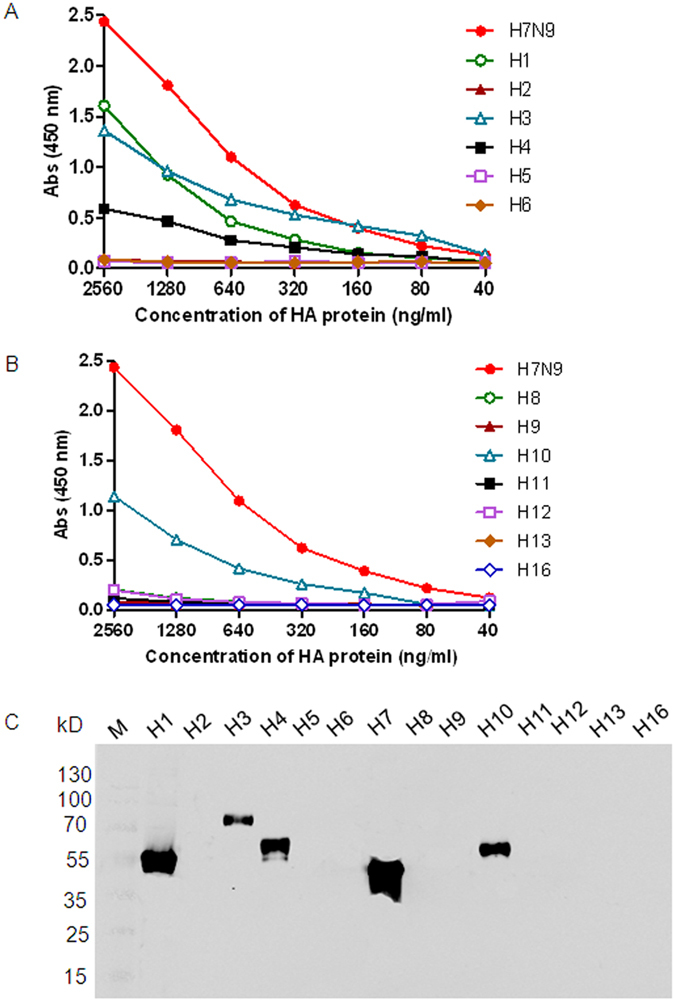
Cross-reactivities between the HA proteins of heterosubtypes of influenza A viruses and antibodies against H7N9. The cross-reactivities were analyzed using ELISA (**A,B**) and Western blot (**C**) with recombinant HA proteins of H1, H2, H3, H4, H5, H6, H8, H9, H10, H11, H12, H13, and H16 as antigens. HA proteins were two-fold diluted with a starting dilution of 2,560 ng/ml.

To evaluate whether there was two-way cross-reactivity between H7N9 and heterosubtype influenza viruses, purified HAs of 13 influenza A subtypes (H1 - H6, H8 - H13, and H16) were tested with antisera against recombinant H7N9 trimeric HA protein by ELISA and Western blot analyses. The ELISA results showed that antisera against Anhui/1 HA also recognized the HA proteins of H1, H3, H4, and H10 at a low to medium level (Fig. 3A,B). Furthermore, Western blot results showed that antisera against Anhui/1 reacted with HA proteins of H1, H3, H4, and H10 (Fig. 3C).

Taken together, the above results suggest that diverse levels of cross-reactivities exist between H7N9 and heterosubtypic viruses. These cross-reactivities are two-way for H3, one-way for H1 and H10, two-way at a low level for H4, and one-way at low level for H9 and H13 subtypes. Importantly, we found that H7N9 HAs were recognized by antiserum against H3. At the same time, H3N2 HAs were recognized by antisera against A/Anhui/1/2013 H7N9. Thus, it is evident that two-way cross-reactivity was observed between H3 and H7N9. Therefore, the serological assays for H7N9 infection need to consider the cross-reactive response of H7N9 with seasonal influenza strains of H3N2.

### Cross-neutralizing antibody responses among influenza A viruses

**Figure 4:**
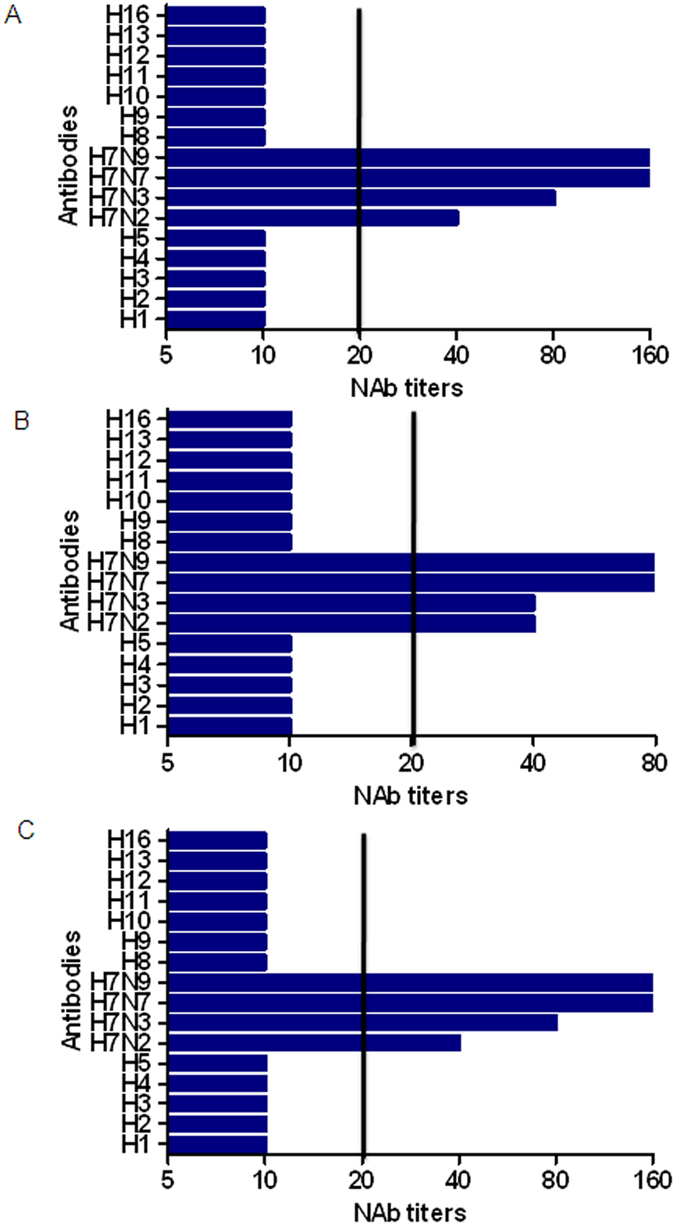
Cross-neutralization of the divergent H7 subtype and heterosubtypes of influenza A viruses to H7N9 virus. The microneutralization (MN) assay was performed using the H7N9 virus strains of Anhui/1 (**A**), Shanghai/1 (**B**), and Shanghai/2 (**C**) and serial dilutions of mouse and rabbit HA antisera. The concentrations of antisera were determined as a series of two-fold dilutions starting with 1:10. Sera with titers ≥20 were considered neutralizing antibodies (NAbs)-positive for H7N9.

HA protein is the major immunogen of vaccination as it can elicit neutralizing antibodies (NAbs). We therefore carried out microneutralization (MN) assays to determine whether the HA proteins of divergent H7 subtypic and heterosubtypic influenza viruses could elicit cross-NAbs to the H7N9 virus. For this purpose, we tested mouse antisera against H7N9, H7N2, H7N3, and H7N7 and representative antisera of H1, H2, H3, H4, H5, H8, H9, H10, H11, H12, H13, and H16 subtypes against Anhui/1, Shanghai/1, and Shanghai/2 isolates. For antisera against the Anhui/1 HA, the NAb titers were 160, 80, and 160 for the Anhui/1, Shanghai/1, and Shanghai/2 isolates, respectively (Fig. 4A–C).

We then tested whether antisera against HA proteins of H7N2, H7N3, and H7N7 could induce cross-reactive NAbs and found that mouse antisera against HA protein of H7N2, H7N3, and H7N7 showed cross-reactive neutralizing activities with the Anhui/1, Shanghai/1, and Shanghai/2 isolates (Fig. 4A–C). These data suggest that common neutralizing epitopes are recognized by mouse antisera against HA proteins of H7N9, H7N2, H7N3, and H7N7 following infection with the Anhui/1, Shanghai/1, and Shanghai/2 isolates. However, no cross-neutralization of the Anhui/1, Shanghai/1, and Shanghai/2 isolates was observed with HA antisera against H1, H2, H5, H8, H9, H11, H12, H13, H16 (group 1), H3, H4, and H10 (group 2). These results suggest that these antisera have no cross-neutralizing activity against H7N9, although some of them show cross-reactivity with H7N9 HA proteins.

### The HA2 subunit of HA mediates cross-reactivity

**Figure 5:**
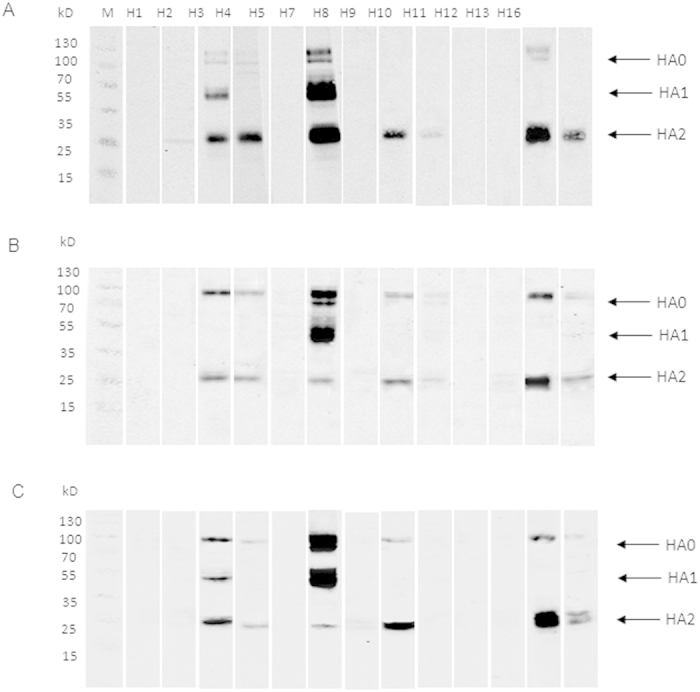
Identification of the cross-reactive regions by Western blot. The lysates of MDCK cells infected with Anhui/1 (**A**), Shanghai/1 (**B**), and Shanghai/2 (**C**) isolates at an MOI of 0.1 were harvested 48 h post infection and probed with antisera to HA proteins of H1, H2, H3, H4, H5, H8, H9, H10, H11, H12, H13, and H16. Antisera against H7N9 HA were used as positive control.

To analyze whether cross-reactivity was mediated through HA1 or HA2 subunits, we infected separate preparations of MDCK cells with Anhui/1, Shanghai/1, and Shanghai/2. The crude cell lysates were used in Western blot analysis with antisera against HA proteins of H1, H2, H5, H8, H9, H11, H12, H13, H16 (all of which belong to group 1), H3, H4, and H10 (all of which belong to group 2) (Fig. 5). The Anhui/1-infected cell lysates showed immunoreactivity with H7N9 HA antiserum at both the HA1 and HA2 subunits (Fig. 5A). The Anhui/1-infected cell lysates also reacted with antisera against HA proteins of H3, H4, H9, H13, and H16 at HA2 subunit (Fig. 5A). Similar results were obtained using the lysates from cells infected with either Shanghai/1 or Shanghai/2 (Fig. 5B,C). These findings suggest that the cross-reactivities between H7 and heterosubtype influenza viruses are mainly mediated through the HA2 subunit.

## Discussion

In this study, we showed that there is cross-reactivity between H7N9 and other subtypes of influenza viruses at diverse levels. These results raise concerns of potential interference from other influenza virus subtypes in the serologic diagnosis of H7N9 infections, especially with the seasonal influenza virus, H3N2. Furthermore, we show that antisera against H7N9, H7N2, H7N3, and H7N7 can effectively neutralize two distinct H7N9 strains, suggesting that H7N9 and divergent H7 HA proteins may serve as potential vaccine candidates for two distinct H7N9 strains.

The emergence of H7N9 and the lack of herd immunity in human populations make this virus a considerable threat to human health. Even though there is no evidence yet of sustained person-to-person transmission of H7N9, the possibility of limited human-to-human spread cannot be excluded^14^,^15^, and continued active surveillance is needed. Although real-time RT-PCR assays have been widely accepted for the diagnosis of H7N9 infections using respiratory specimens^1^,^14^, asymptomatic or mild infections due to contact with infected patients or exposure to live poultry often escape such detections (http://www.who.int/influenza/human_animal_interface/influenza_h7n9/WHO_H7N9_review_31May13.pdf?ua=1). In order to monitor the spread of the virus, it will be critical to perform serologic diagnoses using serum samples collected from infected patients, individuals with contact to patients, individuals with asymptomatic and mild infections, and even the general population. In this study, we found that there is two-way cross-reactivity within group 2 influenza viruses with clear cross-reactivity with the H3 subtype seasonal influenza virus and relatively weak cross-reactivity with H4. However, there is no, or only one-way, cross-reactivity between H7N9 and some group 1 influenza viruses with clear cross-reactivity with the H1 subtype seasonal influenza virus. Our study corroborates previous reports on seasonal influenza viruses that showed that individuals infected with seasonal influenza viruses can have cross-reactive antibodies against HA proteins of different strains and even different subtypes^16^,^17^,^18^. Our results suggest that the serological assays for H7N9 infection need to take into consideration the cross-reactive response of H7N9 with heterosubtypic influenza viruses, especially with seasonal influenza strains. Thus, developing a combination of serological assays to comprehensively evaluate the antibody responses against the H7N9 virus is needed^19^.

The emergence of H7N9 underlines the need to better understand the cross-reactivity between H7N9 and divergent H7 subtypic- and heterosubtypic influenza A viruses for the development of an H7N9 pre-pandemic vaccine. Our results indicate that there are strongly cross-reactive epitopes between H7N9 and H7N2, H7N3, and H7N7, all of which belong to the H7 subtype. There are also cross-reactive epitopes between H7N9 and multiple heterosubtypic viruses. The cross-reactive epitopes identified by ELISA and IFA are conformational or linear epitopes. However, the targeted proteins are denatured during the sample preparation prior to the Western blot assay, and the cross-reactive epitopes identified by Western blot assay are linear epitopes. These cross-reactive epitopes, including conformational and linear epitopes, will need to be confirmed in future studies.

There are cross-reactivities between H7N9 and other subtypes of influenza viruses, and between H7N9 and divergent H7 subtype at diverse levels. However, some, but not all, cross-reactive antibodies have a cross-protection role. Our results suggest that antisera against HA proteins of H7N2, H7N3, and H7N7 can neutralize the H7N9 isolates of Anhui/1, Shanghai/1, and Shanghai/2. This cross-neutralization is in agreement with a recent report by Krammer *et al*., which demonstrated strong cross-reactivity between H7N1 and H7N9 using sera from an H7N1 human vaccine trial^20^. These cross-reactive antibodies could improve *in vivo* virus clearance in a passive transfer H7N9 challenge mouse model. It has also been reported that existing H7 vaccine candidates based on divergent strains offer protection from an H7N9 challenge in mice^21^. Our results, together with the work from others^20^,^21^, suggest that the existing H7 vaccine candidates, such as H7N1, H7N3, and H7N7, have potential applications in the event of an H7N9 pandemic. In addition, phylogenetic analysis has shown that H7N9 is divergent and at least two distinct strains have been found (Anhui/1 and Shanghai/1)^22^. This raises concern about the breadth and efficacy of neutralizing antibodies induced by H7 HA. However, our data show that HA proteins of H7N9, H7N2, H7N3, and H7N7 induced high titers of antibodies and potently neutralized the three H7N9 isolates: Anhui/1, Shanghai/1, and Shanghai/2. These results suggest that the HA proteins of H7N9, H7N2, H7N3, and H7N7 can induce broadly neutralizing antibodies against diverse H7N9 isolates, a critical aspect for H7N9 vaccine design.

In summary, we have evaluated the cross-reactivity between the H7N9 virus and group 1 and 2 influenza A viruses. Our results suggest that the serologic diagnosis for H7N9 infection needs to take into consideration the cross-reactivity of H7N9 with other subtype influenza viruses. Antisera against HA proteins of H7N9, H7N2, H7N3, and H7N7 showed broadly neutralizing activities for diverse H7N9 isolates, suggesting their potential application for vaccine design against H7N9 infections.

## Materials and Methods

### Reference viruses, HA proteins, and antisera

The two distinct H7N9 virus strains, including A/Anhui/1/2013 H7N9, A/Shanghai/1/2013 H7N9, and A/Shanghai/2/2013 H7N9 isolates (hereafter Anhui/1, Shanghai/1, and Shanghai/2, respectively), were cultured on Madin-Darby canine kidney (MDCK) cells and grown in Dulbecco’s modified Eagle medium (DMEM; Hyclone, South Logan, UT) with 10% fetal bovine serum (Hyclone). Sequencing analysis indicates that Anhui/1 and Shanghai/ 2 isolates are highly similar and belong to one strain, whereas Shanghai/1 isolate belong to another strain^1^.

The HA genes of H7N9 Anhui/1, H7N2, H7N3, and H7N7 (Table 1) were synthesized by Sangon Biotech (Shanghai, China). Recombinant HA proteins of H7N9, H7N2, H7N3, and H7N7, which contain a different trimerization domain (GCN4pII) and His-tag, were prepared using a baculovirus expression system (Invitrogen, Carlsbad, CA) as previously described^23^. The recombinant proteins were purified using HisTrap HP and Superdex-200 gel filtration chromatography (GE Healthcare, Waukesha, WI). Following vaccination, mouse polyclonal antisera against the HA proteins of H7N9, H7N2, H7N3, and H7N7 were generated as previously described^24^. This study was carried out in strict accordance with the Chinese government’s animal experiment regulations. All the animal experiments were performed in the facilities of the Institute of Laboratory Animal Sciences (ILAS), Chinese Academy of Medical Sciences (CAMS). All the experimental procedures were approved and supervised by the Animal Protection and Usage Committee of ILAS, CAMS.

The HA proteins of 13 subtypes of influenza virus, including H1, H2, H3, H4, H5, H6, H8, H9, H10, H11, H12, H13, and H16 (Table 1), were expressed in HEK-293 cells or in a baculovirus-insect cell system and purified using a 6× His-tag (Sino Biological, Beijing, China). The hemagglutinin activities of the recombinant HA proteins were confirmed using the Octet system (ForteBio, CA) and the hemagglutination assay (data not shown). The antibodies against HA proteins of H1, H2, H3, H4, H5, H7N9, H8, H9, H10, H11, H12, H13, and H16 were produced in rabbits immunized with the corresponding purified recombinant HA proteins and the antibodies were purified by Protein A affinity chromatography (Sino Biological).

### Western blot

Three different preparations of MDCK cells were infected with Anhui/1, Shanghai/1, and Shanghai/2 at a multiplicity of infection (MOI) of 0.1 for 48 h. The cells were collected and lysed in RIPA buffer (Roche, Indianapolis, IN) and incubated at 100 °C for 10 min. Western blot assays were performed as described with mouse or rabbit HA antisera as primary antibodies^25^.

### Enzyme-linked immunosorbent assay (ELISA)

Recombinant HA proteins of H7N9 Anhui/1, H7N2, H7N3, and H7N7 (250 ng/ml) or two-fold serially diluted heterosubtype HA proteins (starting with 2560 ng/ml) were used as coating antigens in ELISA tests as previously described^7^.

### Indirect immunofluorescence assay (IFA)

Three different preparations of MDCK cells were infected with H7N9 Anhui/1, Shanghai/1, and Shanghai/2 at a MOI of 0.1 in 96-well plates for 48h. The cells were fixed with 4% formaldehyde in PBS for 30 min at room temperature for further IFAs as previously described^26^. The primary antibodies used were mouse or rabbit serum against HA proteins. The plates were scanned, and the images were collected with an Operetta High Content Screening system (PerkinElmer, Waltham, MA). Images were then analyzed with Harmony software (PerkinElmer).

### Microneutralization (MN) assay

MN assays were performed as described previously^27^ using H7N9 virus isolates of Anhui/1, Shanghai/1, and Shanghai/2 and serial dilutions of mouse and rabbit HA antisera (starting dilution of 1:10).

### Hemagglutination inhibition (HI) assay

Ferret antisera against H1N1 virus (A/Sichuan/01/2009 H1N1pdm), H3N2 virus (A/Fujiantongan/196/2009), H5N1 virus (A/HongKong/5052/07-RG), and in-house mice antisera against H7N9-, H7N2-, H7N3-, and H7N7- HA were used in HI assays. The HI assays were performed using an H7N9 Anhui/1 isolate in V-bottom 96-well plates with 1% horse erythrocytes according to the World Health Organization protocol (www.who.int/influenza/gisrs_laboratory/cnic_serological_diagnosis_hai_a_h7n9.pdf).

## Additional Information

**How to cite this article**: Guo, L. *et al*. Cross-reactivity between avian influenza A (H7N9) virus and divergent H7 subtypic and heterosubtypic influenza A viruses. *Sci. Rep.* **6**, 22045; doi: 10.1038/srep22045 (2016).

## Supplementary Material

Supplementary Information

## Figures and Tables

**Table 1:** Characteristics of influenza virus HA genes used in this study.

HA genes	Strain	GenBank/GISAID accession no.
H7N9 HA	A/Anhui/1/2013	EPI439507
H7N2 HA	A/New York/107/2003	EU587368
H7N3 HA	A/chicken/British Columbia/GSC_human_B/04	AY646078
H7N7 HA	A/Netherlands/219/03	AY338459
H1 HA	A/California/04/2009	GQ117044
H2 HA	A/Canada/720/05	DQ009917
H3 HA	A/Brisbane/10/2007	KM821339
H4 HA	A/mallard/Ohio/657/2002	CY011036
H5 HA	A/Anhui/1/2005	HM172104
H6 HA	A/chicken/Hong Kong/17/77	AJ410547
H8 HA	A/pintail duck/Alberta/114/1979	CY005971
H9 HA	A/Hong Kong/1073/99	NC_004908
H10 HA	A/duck/Hong Kong/786/1979	AB292412
H11 HA	A/duck/Yangzhou/906/2002	DQ080993
H12 HA	A/green-winged teal/ALB/199/1991	CY006007
H13 HA	A/black-headed gull/Netherlands/1/00	AY684886
H16 HA	A/black-headed gull/Sweden/5/99	AY684891
