# Supplementary material for: Cross-reactivity between avian influenza A (H7N9) virus and divergent H7 subtypic- and heterosubtypic influenza A viruses
Source: Sci Rep. 2016 Feb 24;6:22045. doi: 10.1038/srep22045 (PMC4764949; doi:10.1038/srep22045)
Supplement: Supplementary Information [file srep22045-s1.pdf]

Cross-reactivity between avian influenza A (H7N9) virusand divergent H7 subtypic- and heterosubtypic influenza A viruses

Li Guo[1,2#], Dayan Wang[3#], Hongli Zhou[1], Chao Wu[1],Xin Gao[1], Yan Xiao[1], Lili Ren[1,2],Gláucia Paranhos-Baccalà[4], YuelongShu[3], Qi Jin[1,2*]&Jianwei Wang[1,2*]

[1]MOH Key Laboratory of Systems Biology of Pathogens and Christophe Mérieux Laboratory, IPB, CAMS-FondationMérieux, Institute of Pathogen Biology (IPB), Chinese Academy of Medical Sciences (CAMS) & PekingUnion Medical College, Beijing, P. R. China

[2]Collaborative Innovation Center for Diagnosis and Treatment of Infectious Diseases, Hangzhou, P. R. China

[3]Institute of Viral Disease Control and Prevention, Chinese Center for Disease Control and Prevention, Beijing, P. R.China

[4]Fondation Mérieux, IRF 128 BioSciences Lyon-Gerland, 69365 Lyon, France

[#]These authors contributed equally to this article

*These authors contributed equally to this article

[#]Correspondence to:

Dr. Jianwei Wang

#9 Dong Dan San Tiao, Dongcheng District

Beijing 100730, P. R. China

Tel/Fax: 86-10-67828516

E-mail: wangjw28@163.com

Dr. Qi Jin

#9 Dong Dan San Tiao, Dongcheng District

Beijing 100730, P. R. China

Tel/Fax: 86-10-67828516

E-mail: zdsys@vip.sina.com

SupplementaryFigure Legends

Figure S1. Titers of IgG antibody against HA proteins of H7 subtypes in mouse sera.

Mice were immunized with recombinant HA proteins of H7N9 Anhui/1, H7N2, H7N3, and H7N7. The titers of mouse sera were determined as a series of two-fold dilutions by ELISA using the proteins of H7N9 Anhui/1, H7N2, H7N3, and H7N7 as coating antigens.

Figure S2. Cross-reactivities between H7 subtypes that can infect human by ELISA.

The H7N2 (A), H7N3 (B), and H7N7 (C) HA proteins expressed in insect cells were used as antigens. Mouse antisera against H7N2, H7N3, H7N7,and H7N9 were serially diluted with a starting dilution of 1:2,000 to react with each antigen.

Figure S3. Cross-reactivity between H7N9 HA and antibodies against heterosubtypes of influenza A viruses by immunofluorescence.

MDCK cells infected with Shanghai/1 (A) and Shanghai/2 (B) at an MOI of 0.1 were fixed with 4% formaldehyde and probed with antisera against HA proteins of H1, H2, H3 H4, H5, H8, H9, H10, H11, H12, H13, and H16 with a concentration of 0.5 μg/ml. Antisera against H7N9 HA were used as positive control.

Figure S1

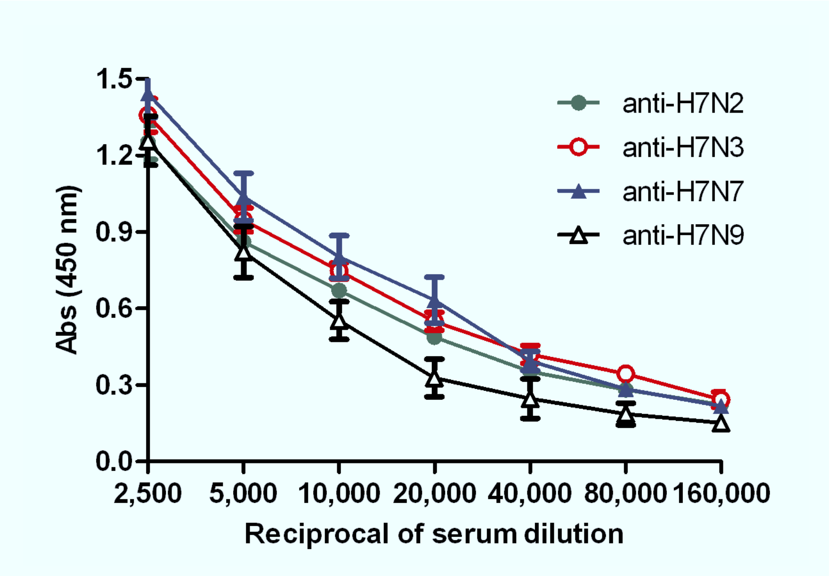

1.5
1.2
0.9
0.6
0.3
0.0
Abs (450 nm)
anti-H7N2
anti-H7N3
anti-H7N7
anti-H7N9
2,500
5,000
10,000
20,000
40,000
80,000
160,000
Reciprocal of serum dilution

Figure S2

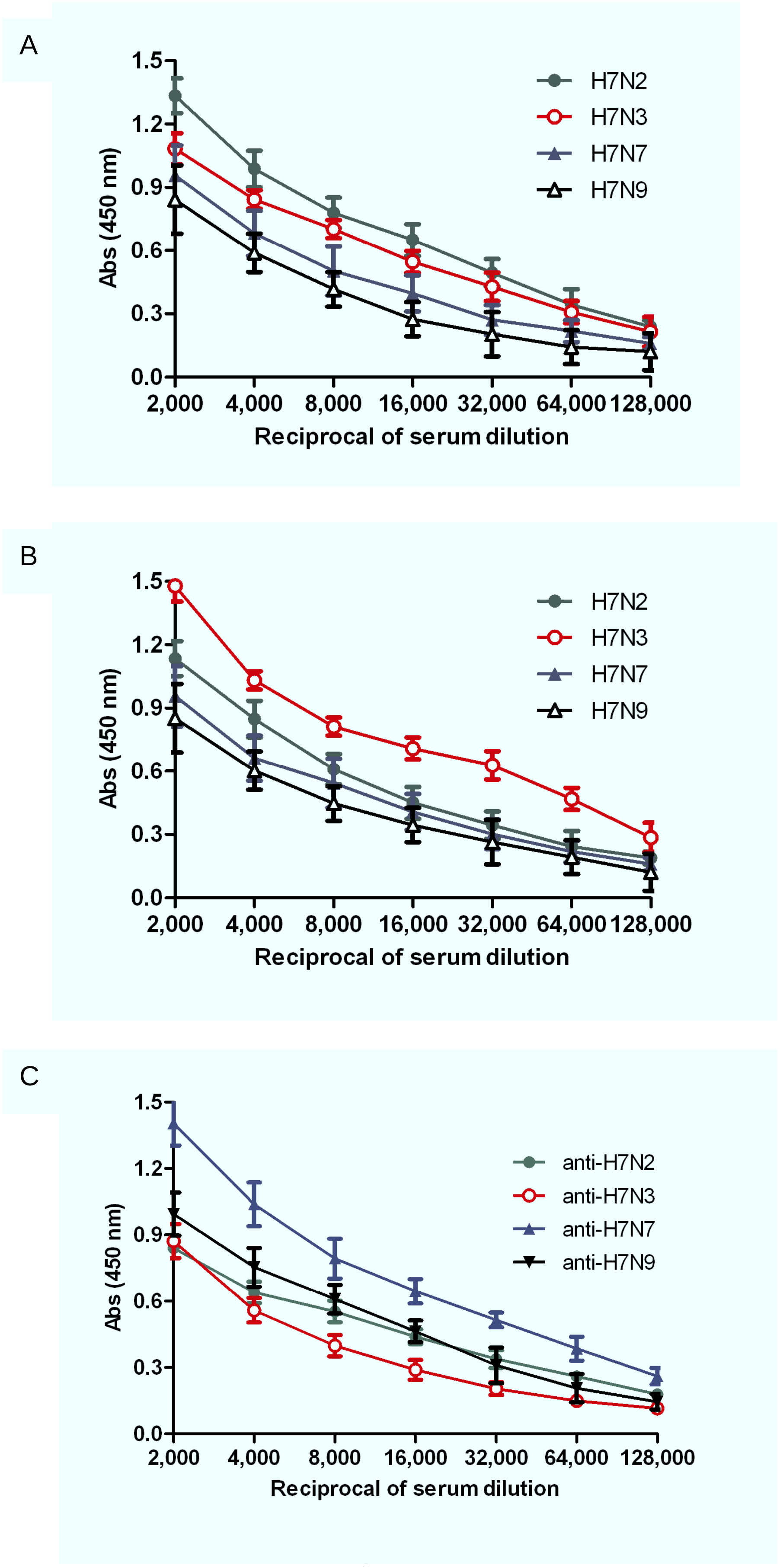
A
1.5
1.2
0.9
0.6
0.3
0.0
Abs (450 nm)
2,000
4,000
8,000
16,000
32,000
64,000
128,000
Reciprocal of serum dilution
H7N2
H7N3
H7N7
H7N9
B
1.5
1.2
0.9
0.6
0.3
0.0
Abs (450 nm)
2,000
4,000
8,000
16,000
32,000
64,000
128,000
Reciprocal of serum dilution
H7N2
H7N3
H7N7
H7N9
C
1.5
1.2
0.9
0.6
0.3
0.0
Abs (450 nm)
2,000
4,000
8,000
16,000
32,000
64,000
128,000
Reciprocal of serum dilution
anti-H7N2
anti-H7N3
anti-H7N7
anti-H7N9

Figure S3

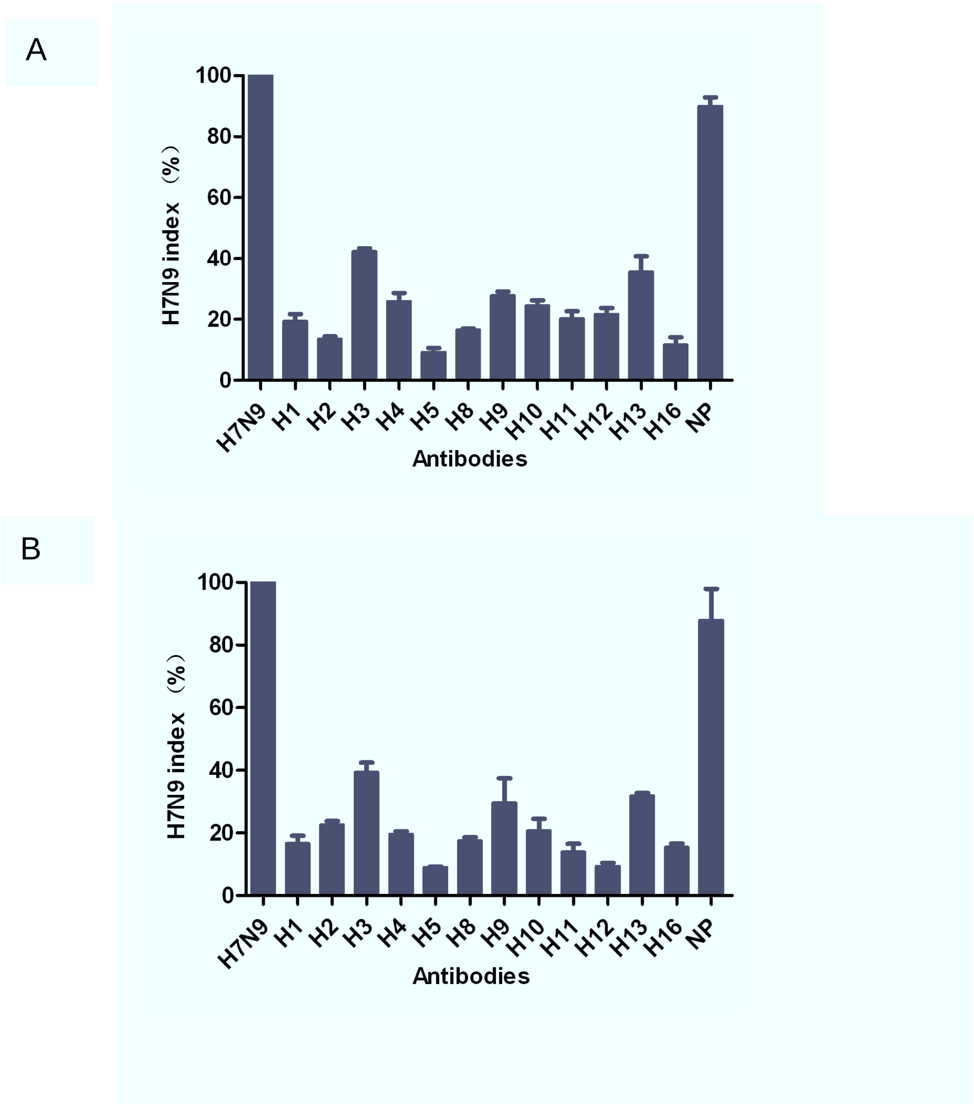
A
100
80
60
40
20
0
H7N9 index （%）
H7N9
H1
H2
H3
H4
H5
H8
H9
H10
H11
H12
H13
H16
NP
Antibodies
B
100
80
60
40
20
0
H7N9 index （%）
H7N9
H1
H2
H3
H4
H5
H8
H9
H10
H11
H12
H13
H16
NP
Antibodies
